# Prospective survey of veterinary practitioners’ primary assessment of equine colic: clinical features, diagnoses, and treatment of 120 cases of large colon impaction

**DOI:** 10.1186/1746-6148-10-S1-S2

**Published:** 2014-07-07

**Authors:** Kyra Megan Jennings, Laila Curtis, John Harold Burford, Sarah Louise Freeman

**Affiliations:** 1 School of Veterinary Medicine and Science, University of Nottingham, College Road, Sutton Bonington, Loughborough, Leicestershire, LE12 5RD, United Kingdom

## Abstract

**Background:**

Large colon impactions are a common cause of colic in the horse. There are no scientific reports on the clinical presentation, diagnostic tests and treatments used in first opinion practice for large colon impaction cases. The aim of this study was to describe the presentation, diagnostic approach and treatment at the primary assessment of horses with large colon impactions.

**Methods:**

Data were collected prospectively from veterinary practitioners on the primary assessment of equine colic cases over a 12 month period. Inclusion criteria were a diagnosis of primary large colon impaction and positive findings on rectal examination. Data recorded for each case included history, signalment, clinical and diagnostic findings, treatment on primary assessment and final case outcome. Case outcomes were categorised into three groups: simple medical (resolved with single treatment), complicated medical (resolved with multiple medical treatments) and critical (required surgery, were euthanased or died). Univariable analysis using one-way ANOVA and Tukey’s *post-hoc* test, Kruskal Wallis with Dunn’s *post-hoc* test and Chi squared analysis were used to compare between different outcome categories.

**Results:**

1032 colic cases were submitted by veterinary practitioners: 120 cases met the inclusion criteria for large colon impaction. Fifty three percent of cases were categorised as simple medical, 36.6% as complicated medical, and 9.2% as critical. Most cases (42.1%) occurred during the winter. Fifty nine percent of horses had had a recent change in management, 43% of horses were not ridden, and 12.5% had a recent / current musculoskeletal injury. Mean heart rate was 43bpm (range 26-88) and most cases showed mild signs of pain (67.5%) and reduced gut sounds (76%). Heart rate was significantly increased and gut sounds significantly decreased in critical compared to simple medical cases (p<0.05). Fifty different treatment combinations were used, with NSAIDs (93%) and oral fluids (71%) being administered most often.

**Conclusions:**

Large colon impactions typically presented with mild signs of colic; heart rate and gut sounds were the most useful parameters to distinguish between simple and critical cases at the primary assessment. The findings of seasonal incidence and associated management factors are consistent with other studies. Veterinary practitioners currently use a wide range of different treatment combinations for large colon impactions.

## Background

Impactions are a common cause of colic [1-3], and occur when ingesta causes obstruction of the digestive tract. They can occur throughout the gastrointestinal tract, but most frequently affect the large colon. The location of the impaction affects both treatment and prognosis. Caecal and small colon impactions have a high rate of surgical intervention, with referral hospital based case studies reporting surgical intervention rates of 43% of 114 cases of caecal impaction [4], and 44% of 84 cases of small colon impaction [5]. Caecal impactions have a high risk of rupture, with a mortality rate of 25% [4]. In contrast, large colon impactions usually have a successful outcome with simple medical treatment, but a small number may require prolonged fluid therapy or surgical treatment [2,6]. Dabareiner and White (1995) reported on a case series of large colon impactions from a referral hospital population, with 16% of cases requiring surgery, and a survival rate of 95% for medical cases, and 58% for surgical cases [2].

A number of risk factors are anecdotally linked with large intestinal impactions; Hillyer *et al.*(2002) identified management factors which were significantly associated with SCOD (Simple Colonic Obstruction and Distension) in a case control study of 76 horses admitted to two UK university referral hospitals [7].

The current published evidence on risk factors, clinical presentation and decision making in large colon impaction cases is based on referral hospital populations [2,7,8], which represent a particular section of the case population. There is a lack of evidence from first opinion practice on the wider population, and on how large colon impaction cases initially present to the veterinary surgeon. The aim of this study was to describe the initial clinical presentation, and the diagnostic tests and treatments used by veterinary practitioners on the primary assessment of horses with large colon impactions.

The study objectives were:

• To describe the history and signalment of large colon impaction cases in a prospective study of the primary presentation of horses with colic to veterinary practitioners.

• To characterise the clinical findings of large colon impaction cases at initial presentation in a prospective study of horses with colic

• To determine how clinical findings of large colon impaction cases on primary presentation relate to final case outcome

• To describe the diagnostic tests and treatments used by veterinary practitioners on the primary assessment of large colon impaction cases.

## Methods

### Study population and data collection

This study reviewed case records relating to impaction cases, obtained from a national survey of the primary presentation of colic cases in the UK, undertaken by the School of Veterinary Medicine and Science, University of Nottingham (http://www.colicsurvey.com). The study was reviewed and approved by the School of Veterinary Medicine and Science Ethics Committee.

The national colic survey was distributed to 850 practices registered with the RCVS in the UK as treating horses, and the launch of the survey was highlighted in the Veterinary Record and Equine Veterinary Education journals, and through the University of Nottingham, RCVS charitable trust and BEVA websites. Veterinary surgeons wishing to participate registered for the survey, and were given a unique reference number to provide confidentiality. They were then asked to submit data on colic cases seen, using an online or paper based recording system. Colic was defined as ‘An incidence of any condition signified by one or more indicators of acute abdominal pain’ [9], and onset of a new case of colic was defined as occurring ‘at least seven days after the end of the previous episode’ [10]. Continuing participation by the veterinary surgeons throughout the study was encouraged by weekly email reminders to submit case information, and regular newsletters on the progress of the study.

The data collection form for each case included open and closed questions covering signalment, history, current management, any health issues, any recent changes in management, clinical presentation and physical examination findings on first presentation, diagnostic approach and findings, and treatments administered on the first assessment, and final case outcomes. A descriptive system was used to evaluate the severity and frequency of kicking, pawing, sweating, flank watching, attempts to lie down and demeanour, based on previous studies [11-13]. Kicking, pawing, flank watching and attempts to lie down were scored as 0: none, 1: occasional, 2: frequent and 3: continuous. Sweating was scored as 0: none, 1: slight, 2: moderate and 3: severe. Demeanour was scored as 0: standing normally/BAR, 1: lower head, no response to auditory stimulus and 2: twitching, agitations and continuous movement. These scores were summed to give maximum severity score of 17. Gastrointestinal sounds on auscultation of each flank quadrant were assessed using descriptors, which were converted to a numerical value (0= absent, 1= reduced, 2= normal and 3= hypermotile); these were analysed for each quadrant of the abdomen (maximum of 3) and summed to calculate an overall numerical score for analysis, with a maximum of 12 [14]. If there was an incomplete data set for any variable, the number of cases with available data was recorded as (n=) for each variable.

### Selection of large colon impaction cases

The data for the impaction cases was retrieved from case records collected for the survey over a 12 month period (October 2012-October 2013). Inclusion and exclusion criteria were used to identify and define the large colon impaction population for this study. An impaction was defined as any case with obstructed lumen of the left ventral large colon or pelvic flexure (including ingesta and sand impactions) and was identified by searching the clinical diagnosis made by the examining veterinary surgeon, and submitted findings of the rectal examination for relevant terms. The complete data sets for each case were reviewed, and cases were excluded if the obstruction was not located in the large colon or if the impaction was considered secondary or associated with other gastrointestinal abnormalities. Final inclusion criteria were horses that were diagnosed with a primary large colon impaction, with positive findings on rectal examination.

If multiple records were submitted for repeated visits for a single case, the physical variables, diagnostic tests and treatments from the first visit only were used in analysis, but the outcome was determined by reviewing all data for that case. Any cases with final outcomes pending or unrecorded were followed up by contacting the veterinary surgeon for further information.

### Categorisation of data

Open questions were used to record the breed of horse, feeding regime, housing, changes in management, current health problems and treatments, and these were categorised into groups based on review of the data and existing literature.

The outcomes of cases were grouped into three categories: ‘simple medical’ cases, ‘complicated medical’ cases and ‘critical’ cases. ‘Simple medical’ cases were classified as those that resolved spontaneously (with no treatment) or with treatment only at initial visit. Horses that resolved with treatment at multiple visits or that were hospitalised for medical treatment were classified as ‘complicated medical’. Horses that required surgery, euthanasia or that died were classified together as ‘critical’ cases.

### Data analysis

The mean, median, mode, range and standard deviation was calculated for each of the continuous variables and percentage frequencies were calculated for all categorical data.

Each variable was assessed to determine whether they followed a normal distribution by creating Q-Q plots and examination of residuals. Data following a parametric distribution is summarised reporting a mean statistic; the median is used in all other cases. Where relevant assumptions were not violated, univariable ANOVA with Tukey’s *post hoc* test was used to compare data for each of the three outcomes. In cases with non-parametric data, a Kruskal-Wallis test with pairwise comparison using Dunn’s correction was used and presented as an adjusted P-value. Chi-squared analysis was used for categorical data. The null hypothesis was rejected at P<0.05. All analyses were performed using SPSS version 21 (IBM Corp, New York).

## Results

### Study population and selection of cases

**Figure 1:**
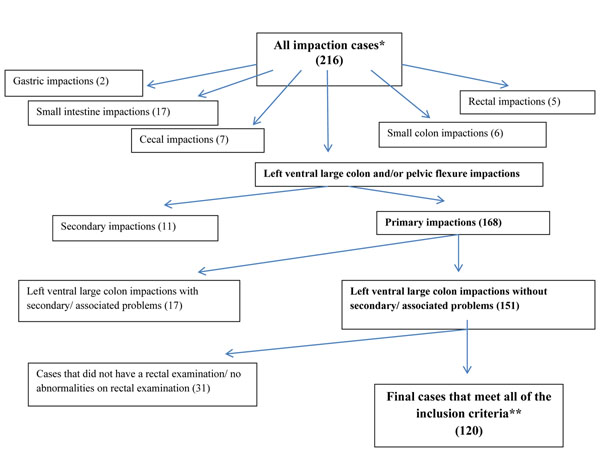
**Identification of primary large colon impaction cases from a database of 1032 colic cases** Flowchart outlining the identification of primary left ventral large colon and/or pelvic flexure impaction cases from a database of 1032 cases in a survey of veterinary practitioner’s primary assessment of colic. * Impaction was defined as obstructed enlarged left ventral large colon and/or pelvic flexure, and was identified by the terms ‘impaction’, ‘obstruction’ or ‘SCOD’ in the database. **Inclusion criteria were cases with a primary impaction of the left ventral large colon and/or pelvic flexure, which were positively identified on rectal examination [2,15,16].

A total of 290 veterinary surgeons registered on the survey, based in 182 different practices. 54% of practices were either first opinion equine or first and second opinion equine, and the remaining practices treated a variety of species in addition to horses. One thousand and thirty two colic cases were submitted over a 12 month period. Two hundred and sixteen impaction cases were identified by using the search terms, and after reviewing the data forms for each, 120 cases met the inclusion criteria (Figure 1). Selected cases were submitted by 69 individual veterinary surgeons. Some forms had data missing from specific sections, therefore the number of cases with data are stated as (n=) for each variable analysis where the complete data set were not available.

### Categorisation and distribution of variables

Thirty seven different breeds were recorded, and were categorised into ‘ponies’, ‘warmbloods’, ‘hotbloods’ (e.g. Thoroughbreds and Arabs) and ‘heavy horses’ (e.g. Draft horses). Health problems were categorised into eight different themes (Table 1). Feed responses were categorised into whether or not horses were fed hay, horsehage, straw and concentrate. Housing management was categorised into whether the owner was the primary carer of the horse or not, and whether the horse was stabled, on permanent grass turnout, or a combination of stabling and turnout. Changes in management were categorised into nine different themes (Table 2).

**Table 1:** Frequency of health problems in 120 horses diagnosed with primary left ventral large colon and/or pelvic flexure impaction in a prospective survey of veterinary practitioner’s primary assessment of equine colic.

Health problem	Number (%) of horses with health problems
Current/recent musculoskeletal injury	15 (12.5%)

Chronic/old musculoskeletal injury	9 (7.5%)

Previous colic episode	11 (9.2%)

Endocrine disorder	5 (4.2%)

Laminitis	4 (3.3%)

Heart murmur	2 (1.7%)

Previous strangles	2 (1.7%)

Other	5 (4.2%)

**Table 2:** Change in management occurring prior to colic in 94 cases of primary left ventral large colon and/or pelvic flexure impaction from a prospective survey of veterinary practitioner’s primary assessment of equine colic.

Category of management change	Number (%) of horses with reported management change
No change	40 (42.6%)

Change in diet	14 14.9%)

Box rest	12 (12.8%)

Increased stabling	10 (10.6%)

Other	8 (8.5%)

Reduced exercise	5 (5.3%)

Moved yards	3 (3.2%)

Weather	3 (3.2%)

Change in field/ turned out	2 (2.1%)

The date on which each colic episode occurred was grouped into seasons, defined as winter (December-February), spring (March-May), summer (June-August) and autumn (September-November).

Open questions on treatments were analysed to assess the different combinations of treatments and which specific drugs were used. Treatments were grouped into non-steroidal anti-inflammatory drugs (NSAIDs), sedatives, spasmolytics, oral fluids, intravenous (IV) fluids, laxatives, opioids and electrolytes, and others.

### Case outcomes

Case outcomes were available for 118 cases. Sixty-three cases (53.4%) were simple medical cases (resolved without treatment or with treatment at the initial visit), two of which resolved with no medical treatment. Forty-four cases (37.2%) were complicated medical cases (resolved with multiple visits or hospitalised for medical treatment), and the remaining 11 (9.3%) were critical cases (1 required surgery, 7 were euthanased and 3 died).

### Signalment

The population of horses in the study consisted of 39 hotbloods, 39 warmbloods, 25 ponies and 14 heavy horses (n=117). There were 60 geldings and 60 mares. Age of the horses ranged from 3 – 41 years (median, 10 years), and estimated body weight ranged from 200 – 750kg (mean 480 ± 107kg; n=115). Body condition was reported as thin in 18 cases, moderate in 82 cases and overweight in 15 cases (n=115).

There was evidence of association between sex and outcome (P = 0.049), with fewer mares having complicated medical or critical outcomes. The estimated body weight of cases with complicated medical outcomes was significantly greater than that of simple medical and critical cases (P = 0.044). No statistically significant differences in breed, age or body condition between the different outcomes were found.

### History

Thirty nine of the 120 cases (32.5%) were reported to have previous or concurrent health problems. Five of these had more than one health problem. The most common problems were current / recent musculoskeletal injury, previous colic episode, and chronic / old musculoskeletal injury (Table 1).

The frequency of dental care was recorded in 73 cases: two horses (2.7%) received dental care every 6 months or less, 42 (57.5%) received dental care every 6–12 months, 18 (24.7%) received dental care every 1–2 years and 11 horses (15.1%) did not receive dental care.

The date on which the horse was last given anthelmintics was recorded for 59 horses: 17 (28.8%) horses had been treated in the last month, (three of which had been in the last seven days), 12 (20.3%) had been treated in the last two months and the remaining 30 (50.8%) had been treated more than two months previously.

The frequency of ridden exercise was recorded in 118 cases: 51 (43.2%) of horses were not ridden at all, 23 (19.5%) were ridden 1-2 times a week, 36 (30.5%) were ridden 3-6 times a week and 8 (6.8%) were ridden every day.

Data was provided on type of feed in 104 cases: 77 (74.0%) horses were fed hay, 32 (30.8%) were fed horsehage, 2 (1.9%) were fed straw and 54 (51.9%) received concentrates.

From 50 responses concerning housing, 20 horses (40.0%) were kept at grass, 17 horses (34.0%) were stabled and 13 horses (26.0%) were stabled and turned out. Eighty (86.0%) horses were kept at the owner’s property or on a DIY (‘Do It Yourself’) livery yard and 11 (11.8%) were kept at full livery (n=93).

Ninety-seven case forms provided information regarding changes in management. Of these responses, 57 horses (59%) had experienced a change in management previous to the colic episode, and the most common changes were change in diet, box rest and increased stabling (Table 2).

There was a variation in the incidence of cases in each season, with the largest number of cases (41.2%) occurring during winter. Thirty three cases (27.7%) occurred in spring, 12 cases (10.1%) in summer and 25 cases (21.0%) in autumn. Changes in management occurred more frequently during the winter months, but there was no significant difference in the number of management changes between the seasons.

### Clinical parameters on primary assessment

Based on the pain severity scoring system of 0-17, four horses (3.3%) were reported as having no signs of pain, 81 horses (67.5%) had mild signs of pain (pain score 1 – 5), 31 horses (25.8%) had moderate signs of pain (pain score 6 – 10) and four horses (3.3%) had severe signs of pain (pain score 10+) on initial presentation (Table 3). There were no evidence of differences between the total pain scores for each of the clinical outcomes, nor differences for each individual pain descriptor category. The mean (range) heart rate for all large colon impaction cases was 43 (26-88); heart rate was significantly higher for cases with a critical outcome compared to simple medical and complicated medical cases (Table 3).

**Table 3:** Mean (range) values for clinical variables in 120 horses diagnosed with primary left ventral large colon and/or pelvic flexure impaction from a prospective survey of veterinary practitioner’s primary assessment of equine colic. Cases were categorised by outcome as simple medical (resolved with single treatment), complicated medical (resolved with multiple medical treatments) and critical (required surgery, were euthanased or died).

Clinical variables (n=number of horses)	Mean value (Range)			
	
	All cases of primary large colon impaction	Simple medical cases	Complicated medical cases	Critical cases
Heart rate (beats/minute) (n=119)	43 (26-88)	42 (26-80)*	41 (26-80)*	56 (36-88)*

Respiration rate (breaths/minute) (n=112)	18 (8-70)	17 (8-70)	17 (8-60)	21 (12-60)

Rectal temperature (°C) (n=103)	37.45 (36.0-39.0)	37.44 (36.0-39.0)	37.37 (36.0-38.3)	37.55 (37.0-38.0)

Total pain score (n=120)	4.6 (0-13)	4.5 (0-13)	4.6 (0-13)	6.0 (2-10)

Total gut sounds (n=120)	5.3 (0-12)	5.5 (0-12)**	5.0 (0-12)**	3.4 (0-8)**

* Critical cases were significantly different to simple medical and complicated medical cases (P=0.008)

**Critical cases were significantly different to simple medical and complicated medical cases (P=0.018)

Gastrointestinal sounds were reduced in most cases (median 4 from a scoring system of 0-12), and total summed gastrointestinal sounds were significantly decreased for critical cases compared to simple medical cases (adjusted significance, P=0.025; Table 3). There was evidence for reduced gut sounds in the right upper and left upper quadrants of critical cases compared to simple medical cases (adjusted significance P=0.011 and P=0.049 respectively); however, there was no evidence of difference between the recorded scores for any quadrants between simple medical and complicated medical cases.

There was no evidence for a difference in respiration rate or rectal temperature between the three outcome categories.

Mucous membrane colours were pink in 95% cases and the remaining 5% were red (n=116). Pulse character was described as strong in 88% of cases and weak in 12% (n=100). Capillary refill time was less than 2.5 seconds in all but 3 of the cases (n=118). Critical cases had significantly different mucous membrane colour (P<0.001) and capillary refill time (P=0.002) compared to other cases.

### Diagnostic tests used at primary assessment

The study inclusion criterion was a positive finding of obstructed lumen of the left ventral large colon or pelvic flexure on rectal examination. Findings on rectal examination were recorded as an open text response, and therefore the descriptions varied. Veterinary practitioners reported that the impaction was located in the pelvic flexure in 61 cases (51%), and gas distention was present in 11 horses (9.2%).

Nasogastric intubation was performed in 99 cases; 96 horses had no abnormalities, three cases were reported to have a small volume of reflux. Of the 99 cases which had nasogastric intubation, 81 received oral fluids. In 26 of these cases, the veterinary surgeons stated on the form that the intubation was performed primarily for treatment purposes. Blood samples were collected in 18 cases. In six of these, samples were not analysed, and in five cases, the results were within normal reference ranges. No information was provided by the veterinary surgeons on the specific tests run in the cases with no abnormalities. There were abnormalities in seven cases (one case with increased PCV, one with leucocytosis, one with decreased neutrophils, one with mild anaemia, one with increased albumin, one with increased lactate, and one with increased ACTH and AST relating to existing endocrine disease). A faecal sedimentation test (“sand test) was performed in three cases, two of which were positive. Abdominocentesis was performed in two cases, both of which had no abnormalities. Ultrasound examinations were performed in two cases, one of which had positive findings (flattening of colon with reduced peristaltic activity with hyperechoic luminal material and normal wall thickness). There was no statistically significant difference between the outcome of the case and the number of diagnostic tests performed at the initial examination.

### Treatment used at primary assessment

The main treatment types given were NSAIDs (n=111), oral fluids (n=84), spasmolytics (n=56), sedatives (n=52), laxatives (n=29), opioids (n=18), electrolytes (n=8) intravenous fluids (n=5), and other treatments (n=7). ‘Other’ treatments included anthelmintics, tetanus antitoxin, steroids, controlled feeding and walking out.

Flunixin was the most frequently used NSAID (42.7% of cases), followed by phenylbutazone (33.6%), meloxicam (16.4%) and ketoprofen (7.3%). Detomidine was the most frequently used sedative (49.0%), followed by xylazine (38.8%) and romifidine (12.2%). Hyoscine was the only spasmolytic, and butorphanol the only opioid used. Complicated medical cases and critical cases were significantly more likely to receive IV fluids (P = 0.043) at the first visit. Cases that resolved with medical treatment alone were significantly less likely to receive laxatives (P=0.041) at the first visit.

A total of 50 different combinations of treatments were used at the primary case presentation. NSAIDs and oral fluids, given to 14 horses (14.3%), was the most frequent combination. There was no statistically significant association between the outcome and the number of treatments used on the first presentation.

## Discussion

### Overview of study and limitations of methodology

This prospective study analysed data from 120 large colon impaction cases. The large number of veterinary practitioners that participated in this study meant that the data were obtained from a wide population, and gives a perspective on the assessments and treatments currently used by practitioners on the primary presentation of the case. The cases covered a wide range of breeds, ages and bodyweights reflecting a diverse population. Disadvantages of the methodology are that with many people contributing data, there may be variation in individual veterinary surgeon’s experience, assessment of cases and use of terminology, and that participants may select specific cases for submission to the survey. The individual variation in response was reduced by using predefined scoring system for key aspects of the questionnaire, such as pain and behaviour, and gastrointestinal sounds, based on current literature [11-13]. Open questions were used for components of the questionnaire such as management change, rectal findings and specific treatments due to the wide range of possible answers and to allow categories to emerge from the data rather than being imposed by the research team. There was considerable variation in the numbers of cases submitted by different practitioners, and the study methodology does not provide an assessment of the incidence or prevalence of impaction colic. It does provide data of how cases initially present, and veterinary practitioners’ primary approaches to these cases.

A total of 216 impaction cases were reported in the overall colic survey, however inclusion criteria for this study was primary large colon impactions which were identified on rectal examination [2,15,16]. Definitive diagnosis in colic cases ideally means confirmation at surgery or *post-mortem* examination, which is not an option in most cases. For this case series, rectal examination was used as the ‘gold standard’, as the findings are usually easily identifiable and characteristic [2,15,16]. Cases with negative findings on rectal examination were excluded as the diagnosis could not be confirmed, but this may have excluded large colon impactions which were not palpable. Other types of impaction, such as gastric, caecal and small colon were excluded from analysis as they presented in small numbers and have a different rate of surgical intervention and mortality [4,5]. Impactions can occur secondary to other conditions, such as grass sickness and small intestinal strangulating lesions, and these were excluded on reviewing case details. The rectal findings were recorded as a free text entry, and therefore the total of 51% pelvic flexure impactions only reflects the numbers described as being in this location, other descriptions gave less details, such as ‘large colon’ impaction. Most of the cases (53%) with treatment at the initial visit only or recovered spontaneously (two cases with no medical treatment). Eleven cases were classified as critical, and only two (1.7%) had surgery. The surgical intervention rate is considerably less than that reported in previous studies (16% and 23%; [2,6]). This may reflect the lower severity of disease seen at the primary presentation of cases in this study compared to the hospital population study by Dabareiner and White [2], or the inclusion and exclusion criteria used. Kaneene *et al.*, [6] described their cases as impaction / acute intestinal obstruction, but the inclusion criteria for this category was not described. A number of factors may affect the decision to take a horse to surgery, including the value of the horse, its current use, any concurrent health problems, insurance status and financial constraints or owner’s previous experiences. Consequently, surgical cases were categorised as critical cases alongside cases that were euthanased and died.

### History and signalment

History and signalment are used by veterinary surgeons to prioritise differential diagnoses and identify possible risk factors and preventative strategies for disease [13]. The identification of significant risk factors requires a case control cohort or cross-sectional study design. Case series, such as this study, provide a lower level of evidence, but can help identify trends which will assist with the design of future studies aimed at identifying risk factors. A number of findings from this study were in agreement with previous studies on impaction and SCOD cases in referral hospital populations, and on non-specific colic cases in the general population. These included the recent changes in diet [7,17-19], an increase in stabling and box rest [2,7] and seasonal variation [20]. A surprisingly large number of horses (n=51) in this study were not ridden; this trend has not been reported previously, and warrants further research to determine whether this is a significant risk factor. Orton *et al.* (1985) showed that exercise can affect gastrointestinal function by decreasing the dry matter digestibility of feed and decreasing mean retention time [21]. The findings of this study, and evidence from previous studies on the effect of exercise and the increased risk associated with box rest [2,7,21] suggest that activity levels may be an important factor in the aetiology of large colon impactions in the horse.

In agreement with other studies, large colon impactions were not limited to a specific breed or age range [2]. There were significant associations between gender and outcome, and bodyweight and outcome; there was no obvious explanation for this difference, and neither association has been reported previously. Univariable analysis was used, and therefore this study did not account for confounding factors.

### Clinical signs

Clinical variables, such as mean heart rate and respiration rate were slightly above normal ranges [13] (Table 3), but similar to those reported in case series of impactions from referral hospitals [2,8]. Heart rate, mucous membrane colour and capillary refill time at the primary presentation were significantly different for critical cases compared to other outcomes. Cardiovascular parameters are widely used as indicators of disease severity [22-25], but this is the first study to report that significant differences are present on the first or initial examination of some patients. This will however be significantly influenced by the severity and duration of the problem, and the owner’s decision to call the veterinary surgeon. In this study, there was considerable individual variation and overlap between different outcome groups with horses in the mild medical category with heart rates of 80 bpm, and one horse with an initial heart rate of 36bpm undergoing surgical treatment, which means that the sensitivity and specificity of heart rate alone at the primary assessment is likely to be low. This may be due to variations in duration of the problem and the progression of the disease at the time of the first presentation to the veterinary surgeon.

A reduction in gut sounds was a common clinical presentation, with 75.8% of cases having reduced gut sounds in at least one flank quadrant, consistent with the study by Dabareiner and White [2]. In this study, critical cases had significantly decreased gut sounds overall compared to medical cases, and reduced gut sounds in the upper left and upper right quadrants, suggesting that both overall and localised changes may be valuable in decision making in large colon impaction cases.

The majority of horses (67.5%) showed mild and intermittent signs of pain on this initial examination. Four cases presented due to mild / intermittent signs of colic or dullness, but had no signs of colic on examination by the veterinary surgeon.

The number of horses showing no signs of pain (3.3%) was considerably lower than the previous referral hospital case series (47.6%) [2], which may reflect prior administration of analgesics or a more chronic history in the hospital case series. This study also found no evidence for differences in pain score between the different outcome categories. This suggests that, unlike most other colic conditions, it cannot be assumed that critical large colon impaction cases will show more severe signs of abdominal pain on their initial examination.

### Diagnosis

Rectal examination is reported to be the most useful diagnostic test to confirm a large colon impaction [2,15,16], and was the inclusion criteria for cases in this study. None of the other diagnostic tests consistently produced positive results for an impaction. Nasogastric intubation was frequently performed but was usually associated with treatment administration (81/99 cases). There were positive findings in two out of the three cases in which sand tests were used, and one out of the two cases in which ultrasound was used. This suggests that these tests may contribute to diagnosis of impactions, but due to the small number of cases in which these tests were used, further data from a larger number of cases is required. A number of factors may affect the veterinary surgeon’s decision to undertake additional diagnostic tests, including the horse’s temperament, facilities available, practitioner’s expertise, and any financial constraints. This study suggests that practitioners frequently use rectal examination and nasogastric intubation on the first examination of cases with large colon impaction cases, and rectal examination was the main test that established diagnosis.

### Treatment

There was a wide range of different treatment combinations used in this study, mainly due to the range of different drugs and treatments available. Recent evidence has demonstrated the efficacy of oral fluids for rehydrating ingesta and resolving impactions [16,26]. NSAIDs and oral fluids were used most frequently; spasmolytics and sedatives were also used commonly, whilst laxatives, IV fluids, opioids and electrolytes were seldom used on the primary assessment. This may reflect the influence of recent research on veterinary practitioners’ choice of treatments, but may also reflect a decision to use less expensive treatments (oral vs IV fluids) at the initial presentation.

Some treatments, such as spasmolytics and sedatives, have a temporary inhibitory effect on intestinal motility [27,28], which may be undesirable in impaction cases. They are however commonly used to improve patient co-operation, and patient and veterinary surgeon safety during diagnostic procedures such as rectal examination and nasogastric intubation. Low doses of hyoscine and alpha-2 agonists have a short duration of effect (<30 minutes) [27,29,30], and the majority of practitioners in this study used detomidine and xylazine, which have a shorter duration of action than romifidine [31,32].

Flunixin was the NSAID used most frequently in this study followed by phenylbutazone. Flunixin has a greater analgesic potency and efficacy than phenylbutazone [33], and has no effect on motility which may explain why it was the most common choice.

The large range of different treatment combinations used to treat the 120 cases in this study highlights the variation between veterinary practitioners in their treatment approach. Once again, the current existing evidence on treatment is based on experimental studies and referral hospital case populations, and this study highlights the need for evidence generated by and targeted at front-line practitioners.

## Conclusions

This is the first study of primary presentation of large colon impactions in the UK. It provides the first evidence on how cases present and are initially assessed and treated by veterinary practitioners. The study confirms evidence from previous research on history and management factors, but also raises new questions regarding the association between exercise and impaction colic. The high proportion of cases that did not have ridden exercise has not been previously reported, and warrants further investigation. Heart rate and gut sounds were the most useful clinical parameters to identify critical cases on the initial presentation. Rectal examination and nasogastric intubation were commonly used by practitioners. Nasogastric intubation was usually associated with fluid administration. Other diagnostic tests were used infrequently and provided limited additional information in most cases. The most common treatments used were NSAIDs and oral fluids, consistent with current evidence. The study highlighted a wide variation in specific treatments by practitioners, and the need for further research on the efficacy of different treatment options outside of the referral hospital environment.

## Competing interests

The authors have no competing interests.

## Authors' contributions

SF, JB and LC designed and co-ordinated the national colic survey. LC was the primary researcher running and collating data from the national colic survey. SF, JB, LC and KJ all contributed to the design of the impaction case series study. KJ retrieved data for impaction cases, and reviewed and analysed impaction data for this study. JB and LC contributed to statistical analysis. KJ and SF were primary authors of the manuscript, but all authors contributed to the final version.

## Declarations

Open access publication of this research was funded by the University of Nottingham.

This article has been published as part of *BMC Veterinary Research* Volume 10 Supplement 1, 2014: Selected articles from the Eleventh International Equine Colic Research Symposium. The full contents of the supplement are available online at http://www.biomedcentral.com/bmcvetres/supplements/10/S1. Publication of this supplement has been funded by The British Equine Veterinary Association.
